# Single-Handed Helical Polybissilsesquioxane Nanotubes and Mesoporous Nanofibers Prepared by an External Templating Approach Using Low-Molecular-Weight Gelators

**DOI:** 10.3390/gels3010002

**Published:** 2017-01-01

**Authors:** Jing Hu, Yonggang Yang

**Affiliations:** Jiangsu Key Laboratory of Advanced Functional Polymer Design and Application, Department of Polymer Science and Engineering, State and Local Joint Engineering Laboratory for Novel Functional Polymeric Materials, College of Chemistry, Chemical Engineering and Materials Science, Soochow University, Suzhou 215123, China; 20154209044@stu.suda.edu.cn

**Keywords:** polybissilsesquioxane, supramolecular templates, optical activity, mesoporous, nanotube

## Abstract

Chiral low-molecular-weight gelators (LMWGs) derived from amino acids can self-assemble into helical fibers and twisted/coiled nanoribbons by H-bonding and π–π interaction. Silica nanotubes with single-handed helices have been prepared using chiral LMWGs through sol–gel transcription. Molecular-scale chirality exists at the inner surfaces. Here, we discuss single-handed helical aromatic ring-bridged polybissilsesquioxane nanotubes and mesoporous nanofibers prepared using chiral LMWGs. This review aims at describing the formation mechanisms of the helical nanostructures, the origination of optical activity, and the applications for other helical nanomaterial preparation, mainly based on our group’s results. The morphology and handedness can be controlled by changing the chirality and kinds of LMWGs and tuning the reaction conditions. The aromatic rings arrange in a partially crystalline structure. The optical activity of the polybissilsesquioxane nanotubes and mesoporous nanofibers originates from chiral defects, including stacking and twisting of aromatic groups, on the inner surfaces. They can be used as the starting materials for preparation of silica, silicon, carbonaceous, silica/carbon, and silicon carbide nanotubes.

## 1. Introduction

Low-molecular-weight gelators (LMWGs) have applications in food, cosmetics, and drugs [1,2,3,4,5,6,7]. Among the LMWGs, chiral LMWGs are the most attractive, and they can self-assemble into chiral nanostructures by hydrogen (H)-bonding, static electric interactions, π–π interactions, and hydrophobic/oleophobic association. For example, lipopeptides can self-assemble into twisted and coiled nanoribbons [8,9,10], and glucosides with long hydrocarbon chains can self-assemble into coiled nanoribbons [11,12]. Over the past few decades, much effort has been focused on transferring the chirality of LMWGs to compounds and nanomaterials. It has been reported that chiral gelators can be used as both media and catalysts for chiral compound synthesis [13,14,15]. Shinkai’s group performed pioneering research on sol–gel transcription using LMWG as external templates for nanomaterial preparation [16]. Single-handed helical silica [17,18], Ta_2_O_5_ [19,20], TiO_2_ [19,21], ZrO_2_ [22], CdS [23], and 3-aminophenol-formaldehyde resin nanotubes [24] have been successfully prepared by this external templating approach. With the development of this external templating approach, a cooperation mechanism was found [25]. Based on this mechanism, mesoporous silicas with helical morphologies have been prepared [26,27,28,29].

Bridged polysilsesquioxanes are a family of organic–inorganic hybrid materials [30]. During the last decades, they have been successfully shaped at the nanoscale. Among these nanostructures, helical nanostructures are the most attractive because of their potential applications in asymmetric autocatalysis and enantiomer separation, and optical materials. Helical polysilsesquioxanes can be prepared using achiral surfactants, and chiral defects, surface free energy, shearing forces, and entropy are considered to drive the formation of the helixes [31,32,33,34,35,36]. Nevertheless, it is difficult to control helical polysilsesquioxanes in enantiopure form, even after addition of chiral dopants [37,38,39]. To control the handedness, Moreau’s group performed pioneering research on sol–gel transcription using LMWGs as self-templates [40,41,42,43,44]. Here, we discuss polybissilsesquioxanes with helical morphologies prepared by an external approach using LMWGs [45,46,47,48,49,50,51,52,53,54,55,56,57,58,59].

## 2. Preparation and Formation Mechanism

### 2.1. Single-Handed Helical Mesoporous Nanofibers

**Figure 1:**
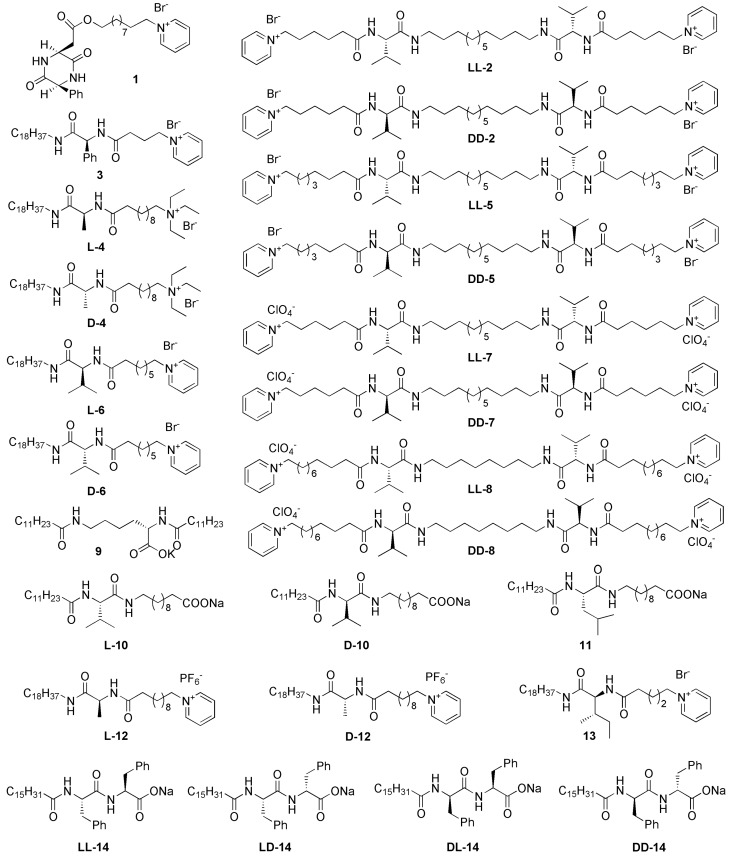
Molecular structures of the low-molecular-weight gelators (LMWGs).

**Figure 2:**
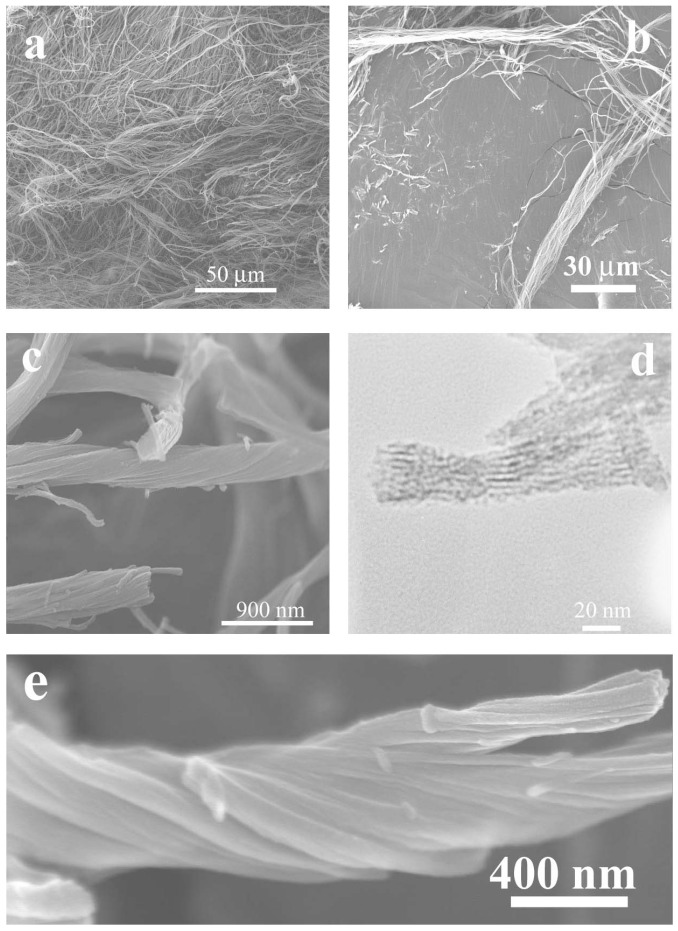
(**a**–**c**) Field-emission scanning electron microscopy (FESEM) and (**d**) transmission electron microscopy (TEM) images of left-handed multiple helical mesoporous 1,4-phenylene-silica nanofibers; (**e**) FESEM image of right-handed multiple helical mesoporous 1,4-phenylene-silica nanofibers. Reproduced with permission from [46]. Copyright 2009 American Chemical Society.

The chiral cationic LMWGs shown in Figure 1 can self-assemble into helical nanofibers or twisted nanoribbons in deionized water or ethanol [45,46,47,48,49,50,51]. Single-handed helical mesoporous polybissilsesquioxane nanofibers, including methylene-, 1,2-ethylene-, 1,2-ethenylene-, 1,4-phenylene-, and 4,4′-biphenylene-bridged nanofibers, have been successfully prepared by sol–gel polymerization using these chiral cationic LMWGs. HCl, NaOH, and ammonium hydroxide are usually used as the catalysts. When the reactions are carried out under acid conditions, sponge-like materials are obtained (Figure 2) [45,46]. The fibrous structure is visible to the naked eye when they are suspended in methanol. Right-handed helical mesoporous 1,4-phenylene-bridged polybissilsesquioxane bundles have been synthesized using **1** or **DD-2**. Left-handed helical 1,4-phenylene-, 1,3-phenylene-, 1,2-ethylene-, and 1,2-ethenylene-bridged polybissilsesquioxane bundles have also been prepared using **LL-2**. These helical bundles can be aligned under shear flow. The pore channels are coiled around the long axes of the nanofibers and do not arrange in a periodic fashion. The mesoporous bundles exhibit nitrogen Brunauer–Emmett–Teller surface areas larger than 500 m^2^/g, and they seem to be a suitable material for catalyst support applications. The X-ray diffraction patterns indicate that 1,4-phenylene-bridged polybissilsesquioxanes synthesized using HCl as the catalyst do not have high molecular-scale periodicity [46,60]. When the reactions are carried out under basic conditions, powders are usually obtained. For example, right-handed twisted 4,4′-biphenylene-bridged mesoporous nanoribbons have been prepared using the self-assemblies of **3** as the template and NaOH as the catalyst [50]. The X-ray diffraction patterns typically show peaks at 2*θ* = 7.46°, 14.76°, 21.09°, 30.83°, and 38.00°, indicating a lamellar structure [61]. The smallest repeat units of the samples arrange with a high degree of order. 

The formation and alignment of the mesoporous polybissilsesquioxane bundles occurs as follows: first, helical gel bundles are constructed by self-assembly of LMWGs and they align under shearing; second, polybissilsesquioxane oligomers penetrate into the gel bundles and adsorb on the surfaces of the helical single-strand gel fibers as a result of electrostatic interactions; third, sol–gel polymerization of the polybissilsesquioxane oligomers occurs; finally, after removing the templates, aligned single-handed helical polybissilsesquioxane mesoporous bundles are obtained. The pore channels should be inner helical and their length can reach 100 μm.

**Figure 3:**
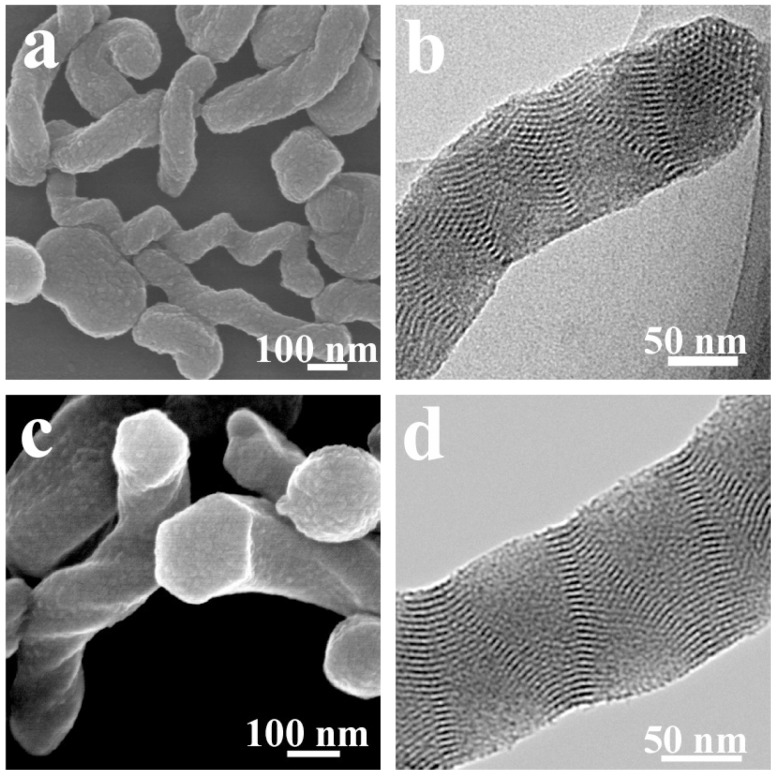
(**a**) FESEM and (**b**) TEM images of helical 1,4-phenylene-bridged polybissilsesquioxane nanorods prepared using **L-4**; (**c**) FESEM and (**d**) TEM images of nanorods prepared using **D-4**. Reproduced with permission from [50]. Copyright 2011 Royal Society of Chemistry.

Because the handedness of polybissilsesquioxane nanofibers can be controlled using chiral LMWGs and the periodic arrangement of the pore channels can be controlled using achiral surfactants, it is preferable to synthesize chiral compounds that can control both handedness and pore arrangement. When sol–gel transcriptions are performed using **L-** or **D-4**, single-handed helical 1,4-phenylene-bridged polybissilsesquioxane nanorods are obtained (Figure 3) [50]. Both the transmission electron microscopy (TEM) images and X-ray diffraction (XRD) patterns indicate a two-dimensional hexagonal symmetry (*p*6*mm*). The formation of the hexagonal structure has been investigated by taking field-emission scanning electron microscopy (FESEM) images after different reaction times. After 1,4-phenylene-bridged bis(silsesquioxane) drops into the reaction mixture, **D-4** and the hybrid silica oligomers coassemble into right-handed helical bundles. With increasing reaction time, hexagonal rods form. Apparently, this structural transition drives formation of single-handed nanorods with periodic mesopores. Based on this structural transition, an artificial frustule has also been prepared [62].

### 2.2. Single-Handed Helical Nanotubes

**Figure 4:**
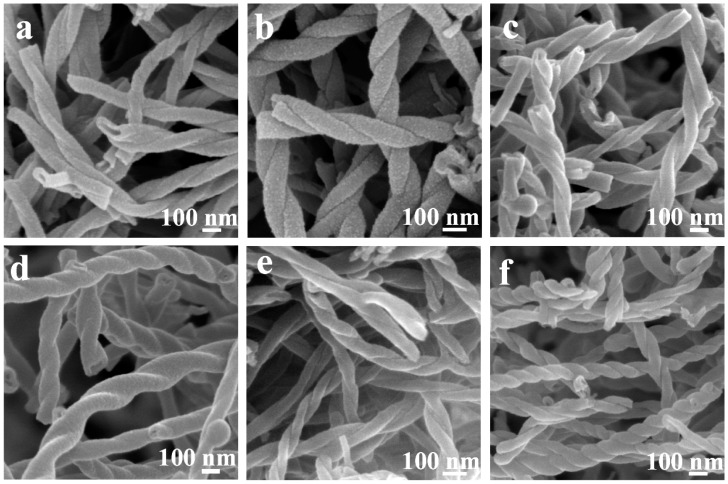
FESEM images of (**a**,**b**) 4,4′-biphenylene bridged polybissilsesquioxane nanotubes; (**c**,**d**) carbon/silica nanotubes; and (**e**,**f**) carbonaceous nanotubes. The samples were prepared using (**a**,**c**,**e**) **LL-8** and (**b**,**d**,**f**) **DD-8**. Reproduced with permission from [47]. Copyright 2013 WILEY-VCH Verlag GmbH & Co. KGaA.

Single-handed helical polybissilsesquioxane tubular nanoribbons and nanotubes have also been prepared using chiral cationic LMWGs [47,48,49]. Generally, the transcriptions were performed in ethanol or a mixture of ethanol and water. Under these conditions, the hydrolysis speed of bis(silsesquioxane) is slow. Formation of the tubular structures can be described as follows. Firstly, helical one-dimensional nanostructures are constructed by the chiral LMWGs in the reaction mixture; secondly, the bis(silsesquioxane) molecules hydrolyze and the formed polybissilsesquioxane oligomers adsorb and polymerize on the surface of the gel fibers; finally, after removing the templates, polybissilsesquioxane nanotubes are obtained. Aromatic ring-bridged polybissilsesquioxane nanotubes have been synthesized in ethanol using compounds **LL**-**7**, **DD-7**, **LL-8**, and **DD-8** (Figure 4) [47,48]. Right-handed helical nanotubes composed of double-coiled nanoribbons have been prepared using **8** in a mixture of ethanol and water [49].

**Figure 5:**
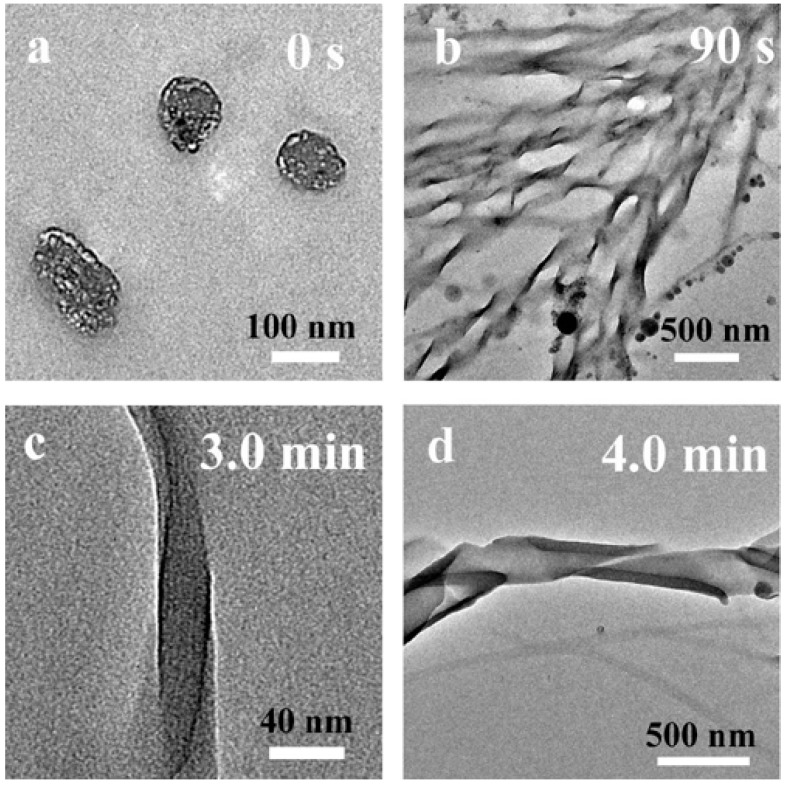
TEM images of the reaction mixture after (**a**) 0 s; (**b**) 90 s; (**c**) 3.0 min; and (**d**) 4.0 min. Reproduced with permission from [52]. Copyright 2008 Royal Society of Chemistry.

For sol–gel transcriptions using anionic LMWGs, addition of co-structure-directing agents is essential [52,53,54,55,56,57,58,59]. The co-structure-directing agents link the anionic self-assemblies by electrostatic interactions and H-bonding, and the bridged silsesquioxanes by covalent bonding. A cooperation self-assembly mechanism was proposed. For example, the morphologies of the self-assemblies of anionic gelator **9** change with addition of 3-aminopropyltrimethoxysilane (APTMS) (Figure 5) [52]. Before addition of APTMS and 1,4-phenylene-bridged bis(silsesquioxane), only nanospheres are identified (Figure 5a). Twisted nanoribbons are then identified at 90 s (Figure 5b). The change of the morphology is due to interactions between gelator **9** and APTMS. Up to now, single-handed helical nanotubes and coiled/twisted tubular nanoribbons have been prepared using anionic LMWGs with the addition of a co-structure-directing agent.

This cooperation self-assembly process is also found in other sol–gel transcriptions. Although the morphologies of the **L-10** and **D-10** self-assemblies are not sensitive to the surrounding conditions (including the pH value, concentration, and ethanol/water volume ratio), handedness inversion of the 4,4′-biphenylene-bridged polybissilsesquioxane nanostructures is found by tuning these conditions [55,59]. Namely, the handedness inversion is driven by cooperation self-assembly. For example, left-handed twisted nanotubes were obtained using **D-10** at pH = 8.05 and a concentration of 5.0 g·L^−1^. However, right-handed twisted nanoribbons were obtained at pH = 11.93 and a concentration of 5.0 g·L^−1^; and right-handed twisted nanotubes were obtained at pH = 9.89 and a concentration of 10.0 g·L^−1^ [55]. When the ethanol/water volume ratio decreases from 1.8/2.2 to 1.5/2.5, polybissilsesquioxanes with opposite handedness are obtained [59]. Not only aromatic ring-bridged polybissilsesquioxane nanotubes, but also ethylene-, ethenylene-, and methylene-bridged ones can be prepared using **11**, indicating that this approach is powerful to control the morphologies of bridged polybissilsesquioxanes [57]. Single-handed twisted nanoribbons have also been prepared using the **14** dipeptide in a mixture of ethanol and water under a basic condition. After carbonization and silica removal, single-handed twisted carbonaceous tubular nanoribbons with optical activity were obtained [58].

**Figure 6:**
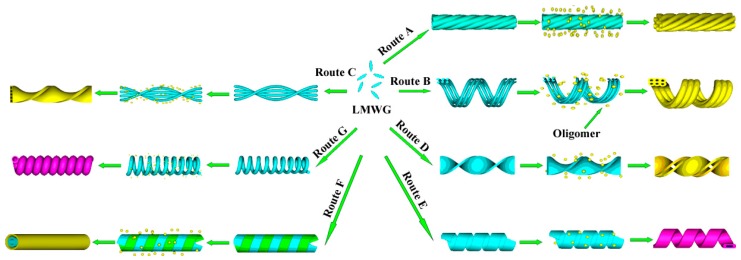
Schematic illustration of formation of single-handed helical polybissilsesquioxane nanostructures. Formation of mesoporous nanofibers (Routes A–C) and that of nanotubes and double-twisted nanoribbons (Routes D–G).

Formation of polybissilsesquioxane nanotubes and mesoporous nanofibers prepared by this external templating approach is summarized in Figure 6. For the preparation of mesoporous nanofibers (Figure 6, Routes A–C), the reactions are usually carried out in water [45]. Under this condition, the rate of hydrolysis of the bis(silsesquioxane) is rapid. The bis(silsesquioxane) oligomers penetrate into the organic self-assemblies and adsorb on the surfaces of single gel fibers. Due to the interactions between LMWG molecule and bis(silsesquioxane) oligomer, the morphologies of the hybrids might change during this step. Mesoporous structures form after the templates are removed. For the preparation of nanotubes and double-twisted nanoribbons (Figure 6, Routes D–G), the reactions are usually carried out in alcohols or a mixture of alcohol and water [47,49]. Under these conditions, the rate of hydrolysis of the bis(silsesquioxane) is slow, and the bis(silsesquioxane) oligomers adsorb on the surfaces of the thick gel nanofibers or nanoribbons. Finally, tubular structures are obtained after the templates are removed. Namely, the hydrolysis rate of bis(silsesquioxane) plays an important role in controlling the tubular and mesoporous structures. 

## 3. Optical Activity of the Aromatic Ring-Bridged Polybissilsesquioxanes

Because aromatic compounds are UV-active, the optical activity of the aromatic ring-bridged polybissilsesquioxanes can be characterized by circular dichroism (CD) and diffuse reflectance CD (DRCD). The CD spectra are usually taken in water or ethanol in suspension states. The optical activity should originate from chiral defects. For example, 4,4′-biphenylene-bridged polybissilsesquioxane tubular nanoribbons prepared using **D-10** and **L-10** exhibit optical activity [59]. When they shrink to form nanoribbons, the inner surfaces merge. Because the chiral defects are partially destroyed, the intensity of the CD signals decreases. For a better understanding of the origin of the CD signals, the CD spectra of 4,4′-biphenylene-bridged bis(silsesquioxane) and 1,4-phenylene-bridged bis(silsesquioxane) dimers have been simulated.

**Figure 7:**
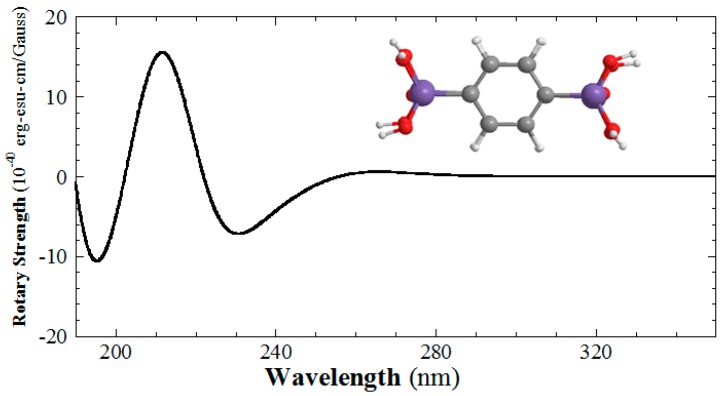
Simulated circular dichroism (CD) spectrum of the 1,4-phenylene-bridged bis(silsesquioxane) dimer at the B3LYP/6-311++G** level with right-handed stacking of phenylene rings.

Time-dependent density functional theory (TD-DFT) at the B3LYP/6-311++G** level has been used to calculate the CD spectrum of the 1,4-phenylene-bridged bis(silsesquioxane) dimer (Figure 7) [51]. When the phenylene groups stack in a right-handed fashion, a positive signal at 260 nm and a negative one at 235 nm are present in the simulated CD spectrum. Electron transfer between the phenylene groups is found. Although the signal at 260 nm is not usually observed in the experimental data, the signal at 235 is strong enough to be observed [51]. The stacking handedness can be determined from the sign of this signal. The relationship between the chirality of 1,4-phenylene-bridged polybissilsesquioxanes at the nanoscale and molecular-scale can be fully understood based on the results of FESEM and CD characterization. Although left-handed coiled 1,4-phenylene-bridged polybissilsesquioxane nanoribbons have been prepared using **L-6** and **13**, they exhibit the opposite optical activity [51]. The results indicated that there is no strong relationship between the chirality at the angstrom level and the handedness at the nanolevel.

**Figure 8:**
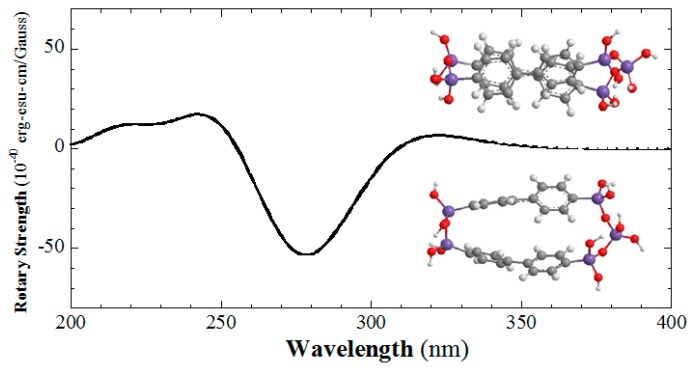
Simulated CD spectrum of the right-handed twisted and stacked biphenylene rings of the 4,4′-biphenylene-bridged bis(silsesquioxane) dimer.

Biphenyls typically exhibit a twisted conformation, with twisting angles of about 30°–45°. The CD spectrum of the 4,4′-biphenylene-bridged bis(silsesquioxane) dimer has been also simulated (Figure 8). The simulated CD signals are present at 319 and 278 nm [49]. If the biphenyl groups stack in right-handedness, the CD signal at 319 nm is positive. If the biphenyl group twists in right-handedness, the CD signal at 278 nm is negative. The simulated CD spectrum is similar as the experimental one [44]. When the distance between neighbor biphenyl groups increases, the CD signals shift to short wavelength. Because biphenyl typically exhibits a twisted conformation and the handedness of the conformation can be revealed by CD spectroscopy, it can be used as a chirality sensor for silicas to reveal the chirality at the angstrom level [49,63].

## 4. TEM, XRD, and N_2_ Sorption Characterizations

The handedness and helical pitch of the single-handed helical structures can be identified using FESEM and electron tomography [64]. The pore architectures are characterized using TEM. For the helical nanofibers, the pore channels are coiled around the long axes (Figure 2d). For the hexagonal rods, the pore channels arrange in a two-dimensional hexagonal structure (Figure 3b,d). Lattice fringes are found in the TEM images of the helically hexagonal rods [50]. The periodicity of the pore channels can be also identified using XRD. For the helical nanofibers, the pore channels do not arrange in a high degree of order. Only one broad diffraction peak is identified in the small angle [46]. When the pore channels arrange in a two-dimensional hexagonal structure, three diffraction peaks are identified at the small angle [50]. The XRD patterns of the aromatic ring-bridged polybissilsesquioxanes usually show several peaks at wide angle, which originate from the lamellar packing of the smallest repeat units and the π–π stacking of the aromatic rings [49,50]. The N_2_ sorption plots of the mesoporous polybissilsesquioxanes usually show typical IV isotherms, indicating rodlike pore channels [50]. The pore diameters are usually about 2–4 nm.

## 5. Other Nanotubes Prepared Using LMWGs

Single-handed helical platinum [65], Ta_2_O_5_ [19,20], TiO_2_ [19,21], ZrO_2_ [22], and CdS [23] nanotubes or tubular nanoribbons have been prepared using sol‒gel transcription. The formation mechanism is similar to that of polybissilsesquioxane nanotubes. Although single-handed twisted platinum and palladium tubular nanoribbons can be prepared using **LL-5** and **DD-5**, gold and silver tubular nanoribbons cannot be obtained. The interactions between the metal ion and the amide group of the gelator have been suggested to drive this structural transcription. All of these single-handed twisted platinum and palladium tubular nanoribbons exhibit optical activity. Based on TD-DFT calculations, the optical activity of these nanotubes has been suggested to originate from chiral defects. The TiO_2_ nanotube could be used as an asymmetric catalyst. The tube should exhibit chirality at the angstrom level [66]. Moreover, although the Ta_2_O_5_ nanotubes prepared using **L-12** and **D-12** are straight, they also exhibit optical activity. The results indicate that the optical activity does not have a strong relationship with the morphology. Organic polymer nanotubes have been also prepared by this approach. Both 3-aminophenol-formaldehyde and 1,3-diamniobenzene-formaldehyde resin nanotubes have been prepared [24]. N-doped carbonaceous nanotubes are obtained by carbonization. Based on TD-DFT calculations, the optical activity is suggested to originate from π–π stacking of the aromatic compounds formed by carbonization.

## 6. Summary and Outlook 

Polybissilsesquioxane nanotubes and mesoporous nanofibers with single-handed helices have been synthesized using self-assemblies of chiral LMWGs as external templates through an external templating approach. They form by a hard templating or cooperation self-assembly process. Because the self-assembly structures of the LMWGs are sensitive to pH value, temperature, and polarity of solvents, both the handedness and the pore architecture of polybissilsesquioxanes can be controlled by tuning the reaction conditions. The optical activity of aromatic ring-bridged polybissilsesquioxanes has been suggested to originate from the π–π stacking and the chiral conformation of aromatic groups. There is no strong relationship between the handedness at the nanolevel and the chirality at the angstrom level. Chiral defects should exist on the inner surfaces of the nanotubes or pore channels. These chiral polybissilsesquioxanes can potentially be used as a chiral stationary phase for enantioseparation. Moreover, carbonaceous and SiC nanotubes can be prepared by carbonization and carbothermal reduction of polybissilsesquioxane nanotubes, respectively. Based on this external templating approach, single-handed helical metal, metal oxide, metal sulfide, and organic polymeric nanotubes can also be prepared. Based on cooperation self-assembly mechanisms, a variety of architectures can be formed, such as artificial frustules and hexagonal rods. Therefore, external templating is a powerful approach for controlling the morphology and pore architecture of nanomaterials.

## References

[1] Wang H., Luo Z., Wang Y., He T., Yang C., Ren C., Ma L., Gong C., Li X., Yang Z. (2016). Enzyme-catalyzed formation of supramolecular hydrogels as promising vaccine adjuvants. Adv. Funct. Mater..

[2] Ren C., Wang H., Mao D., Zhang X., Fengzhao Q., Shi Y., Ding D., Kong D., Wang L., Yang Z. (2015). When molecular probes meet self-assembly: An enhanced quenching effect. Angew. Chem. Int. Ed..

[3] Pont G., Chen L., Spiller D.G., Adams D.J. (2012). The effect of polymer additives on the rheological properties of dipeptide hydrogelators. Soft Matter.

[4] Abul-Haija Y.M., Ulijn R.V. (2015). Sequence peptide-polysaccharide nanostructures by biocatalytic self-assembly. Biomacromolecules.

[5] Pazos E., Sleep E., Pérez C.M.R., Lee S.S., Tantakitti F., Stupp S.I. (2016). Nucleation and growth of ordered arrays of silver nanoparticles on peptide nanofibers: Hybrid nanostructures with antimicrobial properties. J. Am. Chem. Soc..

[6] Guterman T., Kornreich M., Stern A., Adler-Abramovich L., Porath D., Beck R., Shimon L.J.W., Gazit E. (2016). Formation of bacterial pilus-like nanofibers by designed minimalistic self-assembling peptides. Nat. Commun..

[7] Mason T.O., Michaels T.C.T., Levin A., Gazit E., Dobson C.M., Buell A.K., Knowles T.P.J. (2016). Synthesis of nonoequilibrium supramolecular peptide polymers on a microfluidic platform. J. Am. Chem. Soc..

[8] Pashuck E.T., Stupp S.I. (2010). Direct observation of morphological transformation from twisted ribbons into helical ribbons. J. Am. Chem. Soc..

[9] Li Y., Li B., Fu Y., Lin S., Yang Y. (2013). Solvent-induced handedness inversion of dipeptide sodium salts derived from alanine. Langmuir.

[10] Lin S., Li Y., Li B., Yang Y. (2016). Control of the handedness of self-assemblies of dipeptides by the chirality of phenylalanine and steric hindrance of phenylglycine. Langmuir.

[11] Jung J.H., John G., Yoshida K., Shimizu T. (2002). Self-assembling structures of long-chain phenyl glucoside influenced by the introduction of double bonds. J. Am. Chem. Soc..

[12] Jung J.H., Do Y., Lee Y.A., Shimizu T. (2005). Self-assembling structures of long-chain sugar-based amphiphiles influenced by the introduction of double bonds. Chem. Eur. J..

[13] Dawn A., Fujita N., Haraguchi S., Sada K., Shinkai S. (2009). An organogel system can control the stereochemical course of anthracene photodimerization. Chem. Commun..

[14] Jin Q., Zhang L., Cao H., Wang T., Zhu X., Jiang J., Liu M. (2011). Self-assembly of copper(II) ion-mediated nanotube and its supramolecular chiral catalytic behavior. Langmuir.

[15] Rodríguez-Llansola F., Miravet J.F., Escuder B. (2010). Single amino acid based thixotropic hydrogel formation and pH-dependent morphological change of gel nanofibers. Chem. Eur. J..

[16] Ono Y., Nakashima K., Sano M., Kanekiyo Y., Inoue K., Hojo J., Shinkai S. (1998). Organic gels are useful as a template for the preparation of hollow fiber silica. Chem. Commun..

[17] Jung J.H., Ono Y., Hanabusa K., Shinkai S. (2000). Creation of both right-handed and left-handed silica structures by sol−gel transcription of organogel fibers comprised of chiral diaminocyclohexane derivatives. J. Am. Chem. Soc..

[18] Bommel K.J.C., Friggeri A., Shinkai S. (2003). Organic templates for the generation of inorganic materials. Angew. Chem. Int. Ed..

[19] Kobayashi S., Hamasaki N., Suzuki M., Kimura M., Shirai H., Hanabusa K. (2002). Preparation of helical transition-metal oxide tubes using organogelators as structure-directing agents. J. Am. Chem. Soc..

[20] Zhang C., Wang S., Huo H., Huang Z., Li Y., Li B., Yang Y. (2013). Preparation of helical mesoporous tantalum oxide nanotubes through a sol–gel transcription approach. Chem. Asian J..

[21] Zhang C., Wang S., Huo H., Li Y., Li B., Yang Y. (2012). Preparation of helical titania nanotubes using a sol–gel transcription approach. Mater. Lett..

[22] Huo H., Wang S., Lin S., Li Y., Li B., Yang Y. (2014). Chiral zirconia nanotubes prepared through a sol–gel transcription approach. J. Mater. Chem. A..

[23] Zhang C., Huo H., Li Y., Li B., Yang Y. (2013). Preparation of helical CdS nanotubes using a sol–gel transcription approach. Mater. Lett..

[24] Chen H., Li Y., Tang X., Li B., Zhang C., Yang Y. (2015). Preparation of single-handed helical carbonaceous nanotubes using 3-aminophenol-formaldehyde resin. RSC Adv..

[25] Li B., Chen Y., Zhao H., Pei X., Bi L., Hanabusa K., Yang Y. (2008). From branched self-assemblies to branched mesoporous silica nanoribbons. Chem. Commun..

[26] Yang Y., Suzuki M., Owa S., Shirai H., Hanabusa K. (2007). Control of mesoporous silica nanostructures and pore-architectures using a thickener and a gelator. J. Am. Chem. Soc..

[27] Yang Y., Suzuki M., Shirai H., Kurose A., Hanabusa K. (2005). Nanofiberization of inner helical mesoporous silica using chiral gelator as template under a shear flow. Chem. Commun..

[28] Yang Y., Suzuki M., Owa S., Shirai H., Hanabusa K. (2005). Preparation of helical nanostructures using chiral cationic surfactants. Chem. Commun..

[29] Yang Y., Suzuki M., Owa S., Shirai H., Hanabusa K. (2006). Control of helical silica nanostructures using a chiral surfactant. J. Mater. Chem..

[30] Corriu R.J.P. (2000). Ceramics and nanostructures from molecular precursors. Angew. Chem. Int. Ed..

[31] Park S.S., Lee C.H., Cheon J.H., Choe S.J., Park D.H. (2001). Morphological control of periodic mesoporous organosilica with agitation. Bull. Korean Chem. Soc..

[32] Yang S., Zhao L., Yu C., Zhou X., Tang J., Yuan P., Chen D., Zhao D. (2006). On the origin of helical mesostructures. J. Am. Chem. Soc..

[33] Yuan P., Zhao L., Liu N., Wei G., Zhang Y., Wang Y., Yu C. (2009). Periodic mesoporous organosilicas with helical and concentric circular pore architectures. Chem. Eur. J..

[34] Rambaud F., Vallé K., Thibaud S., Julián-López B., Sanchez C. (2009). Hybrid nanofiber growth: One-pot synthesis of functional helicoidal hybrid organic–inorganic nanofibers with periodically organized mesoporosity. Adv. Funct. Mater..

[35] Zhou L., Hong G., Qi L., Lu Y. (2009). Seeding-growth of helical mesoporous silica nanofibers templated by achiral cationic surfactant. Langmuir.

[36] Han Y., Zhao L., Ying Y. (2007). Entropy-driven helical mesostructure formation with achiral cationic surfactant templates. Adv. Mater..

[37] Li Y., Bi L., Wang S., Chen Y., Li B., Zhu X., Yang Y. (2010). Preparation of helical mesoporous ethylene-silica nanofibers with lamellar mesopores on the surfaces. Chem. Commun..

[38] Meng X., Yokoi T., Lu D., Tataumi T. (2007). Synthesis and characterization of chiral periodic mesoporous organosilicas. Angew. Chem. Int. Ed..

[39] Hu Y., Yuan P., Zhao L., Zhou L., Wang Y., Yu C. (2008). Synthesis of enantiomorphic excessive helical mesoporous silicas using chiral molecular dopants. Chem. Lett..

[40] Moreau J.J.E., Vellutini L., Wong Chi Man M., Bied C. (2001). New hybrid organic-inorganic solids with helical morphology via H-bond mediated sol-gel hydrolysis of silyl derivatives of chiral (*R*,*R*)- or (*S*,*S*)-diureidocyclohexane. J. Am. Chem. Soc..

[41] Xu Q., Moreau J.J.E., Wong Chi Man M. (2004). Influence of alkylene chain length on the morphology of chiral bridged silsesquioxanes. J. Sol-Gel Sci. Technol..

[42] Yang Y., Nakazawa M., Suzuki M., Kimura M., Shirai H., Hanabusa K. (2004). Formation of helical hybrid silica bundles. Chem. Mater..

[43] Yang Y., Nakazawa M., Suzuki M., Shirai H., Hanabusa K. (2007). Fabrication of helical hybrid silica bundles. J. Mater. Chem..

[44] Wang Q., Lin S., Qin J., Li Y., Li B., Yang Y. (2016). Helical polybissilsesquioxane bundles prepared using a self-templating approach. Chirality.

[45] Yang Y., Suzuki M., Fukui H., Shirai H., Hanabusa K. (2006). Preparation of helical mesoporous silica and hybrid silica nanofibers using hydrogelator. Chem. Mater..

[46] Wu X., Ji S., Li Y., Li B., Zhu X., Hanabusa K., Yang Y. (2009). Helical transfer through nonlocal interactions. J. Am. Chem. Soc..

[47] Zhang C., Li Y., Li B., Yang Y. (2013). Preparation of single-handed helical carbon/silica and carbonaceous nanotubes using 4,4′-biphenylene bridged polybissilsesquioxane. Chem. Asian J..

[48] Zhang C., Li B., Li Y., Wang M., Yang Y. (2015). Optical activity of SiC nanoparticles prepared from single-handed helical 4,4′-biphenylene-bridged polybissilsesquioxane nanotubes. New J. Chem..

[49] Li B., Xu Z., Zhuang W., Chen Y., Wang S., Li Y., Wang M., Yang Y. (2011). Characterization of 4,4′-biphenylene-silicas and a chiral sensor for silicas. Chem. Commun..

[50] Liu X., Zhuang W., Li B., Wu L., Wang S., Li Y., Yang Y. (2011). Helical periodic mesoporous 1,4-phenylene-silica nanorods with chiral crystalline walls. Chem. Commun..

[51] Li Y., Wang S., Xiao M., Wang M., Huang Z., Li B., Yang Y. (2013). Chirality of the 1,4-phenylene–silica nanoribbons at the nano and angstrom levels. Nanotechnology.

[52] Chen Y., Li B., Wu X., Zhu X., Suzuki M., Hanabusa K., Yang Y. (2008). Hybrid silica tubes with chiral walls. Chem. Commun..

[53] Li H., Li B., Chen Y., Wu X., Zhang J., Li Y., Hanabusa K., Yang Y. (2009). Transfer helix and chirality to 1,4-phenylene silica nanostructures. Mater. Chem. Phys..

[54] Li H., Li B., Chen Y., Zhang M., Wang S., Li Y., Yang Y. (2009). Preparation of chiral 4,4′-biphenylene-silica nanoribbons. J. Chin. Chem..

[55] Li Y., Wang H., Wang L., Zhou F., Chen Y., Li B., Yang Y. (2011). Handedness inversion in preparing chiral 4,4'-biphenylene-silica nanostructures. Nanotechnology.

[56] Wang L., Li Y., Wang H., Zhang M., Chen Y., Li B., Yang Y. (2010). Nanofabrication of helical hybrid silica nanotubes using anionic gelators. Mater. Chem. Phys..

[57] Wang L., Wang H., Li Y., Zhuang W., Zhu Z., Chen Y., Li B., Yang Y. (2011). Formation of helical organic–inorganic hybrid silica nanotubes using a chiral anionic gelator. J. Nanosci. Nanotechnol..

[58] Lin S., Fu Y., Sang Y., Li Y., Li B., Yang Y. (2014). Characterization of chiral carbonaceous nanotubes prepared from four coiled tubular 4,4′-biphenylene-silica nanoribbons. AIMS Mater. Sci..

[59] Liu D., Li B., Guo Y., Li Y., Yang Y. (2015). Inner surface chirality of single-handed twisted carbonaceous tubular nanoribbons. Chirality.

[60] Inagaki S., Guan S., Ohsuna T., Terasaki O. (2002). An ordered mesoporous organosilica hybrid material with a crystal-like wall structure. Nature.

[61] Yang Q., Kapoor M.P., Inagaki S. (2002). Sulfuric acid-functionalized mesoporous benzene-silica with a molecular-scale periodicity in the walls. J. Am. Chem. Soc..

[62] Yan Z., Li Y., Wang S., Xu Z., Chen Y., Li B., Zhu X., Zhu G., Yang Y. (2010). Artificial frustule prepared through a single-templating approach. Chem. Commun..

[63] Xue Z., Zhao Y., Wu L., Li Y., Li B., Wang M., Yang Y. (2013). A chirality indicator for the surfaces of the silica nanotubes. J. Nanosci. Nanotechnol..

[64] Yuan P., Zhao L., Liu N., Wei G., Wang Y., Auchterlonie G.J., Drennan J., Lu G.Q., Zhou J., Yu C. (2010). Evolution of helical mesostructures. Chem. Eur. J..

[65] Cai H., Wang C., Li B., Li Y., Yang Y. (2014). Preparation and characterization of single-handed twisted platinum tubular nanoribbons. Mater. Lett..

[66] Wang S., Li R., Zhang C., Li Y., Li B., Yang Y. (2013). Formation and asymmetric catalysis of C- and 8-shaped titania tubes. Mater. Lett..

